# Linking mitochondrial dynamics and fertility: promoting fertility by phoenixin through modulation of ovarian expression of GnRH receptor and mitochondrial dynamics proteins DRP-1 and Mfn-2

**DOI:** 10.1007/s00424-022-02739-y

**Published:** 2022-08-16

**Authors:** Eman H. Basha, Amira K. B. Eltokhy, Asmaa Fawzy Eltantawy, Nehal A. E. Heabah, Shereef Lotfy Elshwaikh, Yasmeen M. El-Harty

**Affiliations:** 1 Department of Medical Physiology, Faculty of Medicine, Tanta University, Tanta, Egypt; 2 Department of Medical Biochemistry, Faculty of Medicine, Tanta University, Tanta, Egypt; 3 Department of Medical Pharmacology, Faculty of Medicine, Tanta University, Tanta, Egypt; 4 Department of Pathology, Faculty of Medicine, Tanta University, Tanta, Egypt; 5 Obstetrics and Gynecology Department, Faculty of Medicine, Tanta University, Tanta, Egypt

**Keywords:** Obesity, Infertility, Phoenixin, Drp1, Mfn2

## Abstract

Obesity is linked to reproductive disorders. Novel neuropeptide phoenixin demonstrated many therapeutic actions. In this study, we aim to evaluate phoenixin’s potential effect in obesity-induced infertility through modulating mitochondrial dynamics. Ninety adult female rats were divided to 4 groups: (I), fed with normal pellet diet; (II), given phoenixin; (III), fed with high-fat diet. Rats that developed obesity and infertility were divided to 2 groups: (III-A), received no further treatment; (III-B), given phoenixin. Our results showed that phoenixin treatment in obese infertile rats significantly decreased serum levels of insulin and testosterone and ovarian levels of dynamin-related protein1(Drp1),reactive oxygen species ROS, TNF-α, MDA, and caspase-3. Phoenixin treatment also significantly increased serum estrogen progesterone, LH, and FSH together with ovarian levels of GnRH receptor (GnRHR), mitofusin2(Mfn2), mitochondrial transmembrane potential (ΔΨm), and electron transport chain (ETC) complex-I significantly when compared with obese group. Ovarian histopathological changes were similarly improved by phoenixin. Our data demonstrate phoenixin’s role in improving obesity-induced infertility.

**Graphical abstract:**

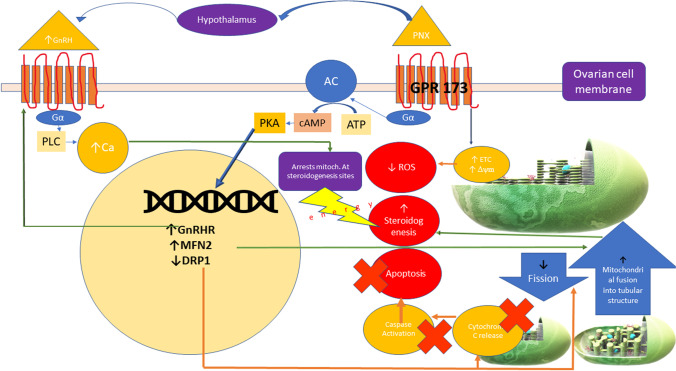

## Introduction

Obesity and overweight have significantly increased around the world, with subsequent detrimental impacts on a variety of human body functions, including reproductive health [35]. Obesity causes intracellular lipid accumulation in many tissues and thus impairs mitochondrial function and lipotoxicity responses, stimulating inflammatory and oxidative stress (OS) pathways that probably culminate in apoptosis [40]. Wu et al. [42] have shown that lipotoxicity is a significant contributor to obesity-induced infertility via alteration of oocyte quality. A high-fat diet also causes hypothalamic inflammatory changes, which eventually alter fertility [6].

Since oxidative stress and apoptosis are hallmarks in obesity, the link to mitochondrial dynamics in such conditions must be considered, especially since the ETC is the primary source of reactive oxygen species (ROS) generation. Mitochondrial dynamics refers to the fusion and fission processes during mitochondrial movement, which are in perfect balance physiologically to allow proper local energy production and distribution needed by each cellular organelle to perform its needed function [7, 9]. The disintegration of this balance results in apoptosis.

Phoenixin (PNX) is a novel neuropeptide first described by Yosten et al. [47] via a bioinformatics approach revealing amidated 14-amino acid peptide and 20-amino acid peptide. The first biological action described for PNX was the potentiation of the GnRH-stimulated release of LH. The stimulation of LH by PNX-14 and PNX-20 is GnRH-dependent, with increased GnRH receptor expression following PNX treatment. It is evident that the GnRH receptor is also expressed in ovarian cells at a 200-fold lower rate than that of the pituitary. GnRH affects ovarian steroidogenesis, proliferation, and survival [23].

The G protein–coupled receptor 173 (GPR173), which belongs to the orphan G-protein-coupled receptor subfamilies, was found to be the mediator of most of the PNX-20’s physiological functions. GPR173 is now known to be widely distributed in various tissues [32]. Despite the finding that PNX was found to be most abundant in multiple hypothalamic nuclei, it was also expressed in other CNS areas, including the brain and the spinal cord, the heart, stomach, esophagus, spleen, kidney, and lung [30, 47].

The widespread PNX expression in various hypothalamic subnuclei denotes that it possibly has a significant influence as a mediator of a wide range of physiological and/or pathophysiological functions. It was found to improve memory formation and retention while also alleviating memory impairment and anxiety [13]. PNX has also been proven one of the neuropeptides responsible for the brain-gut connection with a potent centrally mediated orexigenic effect [31]. Peripherally, PNX has been proposed to play a role in pruritis, mediating its signaling in the peripheral nervous system [5].

With regard to its earlier-described role in the modulation of pituitary gonadotropins, PNX injection was also found to alter reproductive gene mRNA abundance [39], adipogenesis [2], and in vitro human granulosa cell proliferation [25]. In addition, the anti-inflammatory functions of GnRH possibly implicate PNX as an anti-inflammatory compound [48].

Interestingly, Yang et al.[45] provided direct experimental evidence that PNX-20 upgrades neuronal mitochondrial biogenesis which requires further investigations in other organs both in vivo and in vitro to evaluate PNX’s modulatory effect in cases of mitochondrial damage such as obesity.

Also, McIlwraith et al. [22] concluded that nutritional levels and environmental chemicals can possibly modulate the reproductive function through manipulating *Gpr173* expression. This suggests a future therapeutic objective in inflammatory diseases, such as obesity which requires further investigations of the role of PNX in obesity-related fertility impairment.

Considering all this, we hypothesized that PNX could possess a role in the positive modulation of obesity-induced fertility impairment. The current study was conducted to understand the underlying mechanism of PNX’s role in such conditions and its relation to PNX’s effect on mitochondrial functions.

## Material and methods

### Experimental animals

The study was carried out on 90 adult female albino rats weighing (180–220 g). The rats were housed in standard, well-ventilated animal cages at room temperature, with free access to water and food throughout the entire study interval. The maximum number of rats per cage was assigned to three to avoid cage overcrowding or decreased cleanliness. Rats were monitored five times a week for cage aggression or disease signs. All procedures were performed according to the ethical committee of Tanta University (Approval Code Number: 34223/10/20).

### Drugs and chemicals

Phoenixin-14 amide (Asp-Val-Gln-Pro-Pro-Gly-Leu-LysVal-Trp-Ser-Asp-Pro-Phe-NH2) was supplied by Sigma–Aldrich Co, (St. Louis, MO).

Standard rat chow (control rats; 3.8 kcal/g–63.4% carbohydrate, 25.6% proteins, and 11.0% fat).

High-fat diet (obese rats; 5.4 kcal/g–25.9% carbohydrate, 14.9% proteins, and 59.0% fat).

### Animal groups

After 12 days of acclimatization and monitoring of the estrus cycle, the 90 rats showing regular estrus cycle were divided into three groups:

1. Group I: control group (20 rats): The rats were fed on a normal pellet diet for the entire experimental period of 20 weeks.
2. Group II (20 rats): control treated by phoenixin: The rats of this group were given phoenixin-14 once daily at a dose of 100 nmol/g body weight by gastrogavage for ten weeks[44].
3. Group III (50 rats): obese group: The rats were fed on a high-fat diet for 10 weeks to induce obesity. Estrus cycle and body weight were monitored. Rats showing average/low body weight and/or regular estrus cycle were excluded. The remaining rats were then examined for confirmation of obesity-induced infertility via the lipid profile and hormonal state analysis. Forty rats demonstrated disturbed estrus cycle either in irregularity or 2–3 days lengthening. In addition, animal lipid profiles and hormonal states denoted the development of obesity-induced infertility. These rats were furtherly subdivided into two subgroups:
4. Group III-A (20 rats): These rats were given no further treatment and were sacrificed, as mentioned below.
5. Group III-B (20 rats): These rats were given phoenixin-14 once daily at a dose of 100 nmol/g body weight by gastrogavage for 10 weeks [44].

### Blood sample and biochemical assessment of lipid profile, hormonal state

At the end of the experimental period, all rats were weighed, anesthetized with an intraperitoneal injection of pentobarbital (50 mg/kg) [1], sacrificed by cervical dislocation, and blood samples were collected, and then serum was separated by centrifugation at 3000 rpm for 10 min and transferred into clean storage tubes for determining the following parameters: total lipid profile calorimetrically (Biodiagnostic, Egypt), Insulin (Cat# No: ab63820), estradiol (Cat# No: ab108667), progesterone (Cat# No: ab108670), FSH (Cat# No: CSB-E06869r), LH (Cat# No: CSB-E12654r), Testosterone (Cat# No: ab108666), by ELISA kits according to manufacturer’s instructions.

### Preparation of ovarian tissues

Each studied group’s ovarian tissues were randomly divided into three divisions. One division was assigned for biochemical and mitochondrial sample analyses after proper homogenization, and another division was assigned for real-time gene abundance analysis. The third division was assigned for histopathological analyses.

### Biochemical analysis of inflammation, OS, and apoptosis

Tissue samples were collected from anesthetized rats, homogenized in cold phosphate buffer (pH 7.4), and centrifugated at 3000 rpm for 10 min. The supernatant was separated in clean storage plastic test tubes and stored at − 80 °C and was used for immunoassay determination of tumor necrosis factor-α (TNF-α) (Cat# No MBS355371) as a marker of inflammation, caspase-3 activity (Cat# No: ab39401) as a marker of apoptosis; both according to manufacturer’s instruction. Also, OS marker malondialdehyde (MDA) levels were measured per the methodology described by Ohkawa et al. [27].

### Mitochondrial samples analysis

The assigned division of ovarian tissues was homogenized in a mitochondrial buffer, followed by centrifugation for 10 min at 2000 g. Mitochondrial fractions were obtained by centrifuging the supernatants for an additional 20 min at 5000 g. Spectrophotometric methods measured the following mitochondrial parameters: mitochondrial transmembrane potential (ΔΨm) by the method of Maity et al. [20], electron transport chain (ETC) complex-I activity (Cat# No. ab109721).

### Quantitative estimation of GnRHR mRNA abundance, mitochondrial-related protein 1 (Drp1) and mitofusin 2 (Mfn2) relative genes abundance by real-time PCR

#### RNA extraction

Ovarian tissue total RNA isolation was carried out by gene Jet RNA purification kit according to the manufacturer’s instructions (Thermo scientific, # k 0731 USA)0.29. Concentration and purity of total RNA were estimated at the OD260 and OD260/280 ratios, respectively, using Nano Drop spectrophotometer (NanoDrop Technologies, Inc., Wilmington), then stored at − 80 °C.

#### Complementary DNA (cDNA) synthesis

Using reverse transcription, cDNA was then produced from of 5 μg total RNA sample using Reverse Transcriptase (a revert Aid H Minus) (Thermo scientific, # Ep0451). Detection of Drp1 and Mfn2 relative gene abundance was done using the obtained cDNA (as a template) via Step One Plus Real-Time PCR system (Applied Biosystem). The primer sequences have been conceived with Primer 5.0 software (Table 1).

**Table 1:** Primers of Drp1, Mfn2, β-actin, and GnRHR mRNA abundance

Drp1 gene (NCBI GenBank Nucleotide accession # NM_053655.3)	
Forward	5′-GCTAGATGTGCCAGTTCCAGT-3′
Reverse	5′-TGTGCCATGTCCTCGGATTC-3′
Mfn2 gene (NCBI GenBank Nucleotide accession # NM_130894.4)	
Forward	5′-AGTGTCAAGA CCGTGAACCA-3′
Reverse	5′-ACACA TCAGCATCCAGGCAA-3′
β-actin housekeeping gene (NCBI GenBank Nucleotide accession # NM_031144.3)	
Forward	5′-ATCAGCAAGCAGGAGTACGAT-3′
Reverse	5′-AAAGGGTGTAAAACGCAGCTC-3′
GnRHR gene	
Forward	5′-TTCTCATCATGGTGATCTGCAA-3′
Reverse	5′-GCAAATGCAACCGTCATCTTTA-3′

#### The conditions of thermal cycler

First, a 10-min denaturation at 95 °C was followed by 40 to 45 amplification cycles (DNA denaturation for 15 s at 95 °C, annealing for 30 s at 60 °C, and then extension for 30 s at 72 °C). At the end of the last cycle, the temperature increased from 63 to 95 °C for melting curve analysis. The Ct values (cycle threshold) for both (target and housekeeping) genes were estimated, and the relative gene abundance assessment was performed using the 2^−ΔΔCt^ method.

### Histopathological evaluation of ovarian tissues

Ovarian tissues were fixed into 10% buffered formalin and then embedded in paraffin. They were cut into 4-μm-thickness sections using a microtome. Hematoxylin and eosin (H&E) staining was carried out for histopathological evaluation.

### Statistical analysis

The obtained results were represented using the mean ± standard deviation. One-way ANOVA and Tukey’s post hoc test were used to analyze and evaluate the significance. Statistical significance was considered at *p*-values < 0.05. SPSS software (Version 23.0, IMB, NY) was used for statistical analyses. GraphPad Prism software (Version 9.3.1) was used for statistical graphing.

## Results

### Effect of phoenixin treatment on body weight, lipid profile, redox status, and inflammatory response

As shown in Table 2, the results of this work showed that bodyweight is significantly different among the studied groups. The total cholesterol (TC), LDL-c, and TAG showed a significant increase in the obese group as compared to control and PNX-treated groups. HDL-c showed a significant decrease in the obese group as compared to control and PNX-treated groups. TNF- alpha, MDA, and caspase-3 showed significantly elevated levels in the obese group as compared to control and PNX-treated ones. There was no significant difference between the control group and PNX-treated groups in lipid profile, body weight, oxidative stress, inflammatory, and apoptotic markers (Table 2).

**Table 2:** Effect of phoenixin treatment on body weight, redox status, apoptosis, and inflammatory response among the studied groups

parameter	Control group (I)^a^ *N* = 20	Control treated by phoenixin (II) ^b^ *N* = 20	Obese (III)^c^ *N* = 20	Obese treated with phoenixin (IV)^d^ *N* = 20
Body weight (g)	152.8 ± 2.6^(c)^	153.1 ± 4^(c)^	319.8 ± 13.9^(a, b, d)^	154.5 ± 2.4^(c)^
TC (mg/dl)	114 ± 1.2^(c)^	112.3 ± 1.5^(c)^	356.6 ± 1.18^(a, b, d)^	120.3 ± 0.89 ^(c)^
LDL-c (mg/dl)	48.6 ± 1.1^(c)^	46.1 ± 1^(c)^	111.3 ± 1.3^(a, b, d)^	49.4 ± 2^(c)^
HDL-c (mg/dl)	56.6 ± 1.5^(c)^	60.4 ± 4.8^(c)^	16 ± 1.9^(a, c, d)^	56.8 ± 2.6^(c)^
TAG (mg/dl)	67.2 ± 1.3^(c)^	64.6 ± 1.1^(c)^	146.5 ± 5^(a, b, d)^	65.6 ± 1.1^(c)^
MDA (ng/L)	0.9 ± 0.015^(c)^	0.8 ± 0.12^(c)^	2.18 ± 0.71^(a, b, d)^	0.94 ± 0.04^(c)^
TNF alpha (ng/ml)	173.3 ± 3.5^(c)^	170.25 ± 1.3^(c)^	207 ± 8.3^(a, b, d)^	176.8 ± 1.5^(c)^
Caspase 3 (ng/ml)	2.2 ± 0.08^(c)^	1.9 ± 0.13^(c)^	8.5 ± 0.23^(a, b, d)^	3.2 ± 0.09^(b)^

Superscript letters a, b, c, and d denote a statistically significant difference at (*P* < 0.05) using one-way ANOVA with Tukey’s post hoc test

^a^Statistical significance when compared to group I

^b^Statistical significance when compared to group II

^c^Statistical significance when compared to group III

^d^Statistical significance when compared to group IV

### Effect of phoenixin treatment on hormonal profile among the studied groups

Table 3 shows that PNX treatment significantly lowered the obesity-induced significantly high levels of insulin and testosterone. On the other hand, the neuropeptide elevated the level of LH, FSH, estradiol, and progesterone significantly as compared to the obese group. There is no significant difference between hormonal level l among control groups and PNX-treated group.

**Table 3:** Effect of phoenixin treatment on hormonal profile among the studied groups

Hormone	Control group (I)^a^ *N* = 20	Control treated by phoenixin (II)b *N* = 20	Obese (III)c *N* = 20	Obese treated with phoenixin (IV)^d^ *N* = 20
Insulin (ng/ml)	0.9 ± 0.47^(c)^	0.76 ± 0.35^(c)^	9.5 ± 0.49^(a, b, d)^	1.3 ± 0.26^(c)^
Testosterone (ng/ml)	0.8016 ± 0.028^(c)^	0.71 ± 0.08^(c)^	1.6 ± 0.18^(a, b, d)^	0.785 ± 0.04^(c)^
LH (mIU/ml)	0.034 ± 0.004^(c)^	0.041 ± 0.006^(c)^	0.013 ± 0.005^(a, b, d)^	0.041 ± 0.005^(c)^
FSH (mIU/ml)	0.087 ± 0.005^(c)^	0.091 ± 0.0056^(c)^	0.028 ± 0.01^(a, b, d)^	0.083 ± 0.012^(c)^
Estradiol (Pg/ml)	19.44 ± 0.3^(c)^	20 ± 0.49^(c)^	7.36 ± 0.23^(a, b, d)^	18.58 ± 0.27^(c)^
Progesterone (Pg/ml)	37.71 ± 0.5^(c)^	38.19 ± 0.95^(c)^	12 ± 2.7^(a, b, d)^	36.4 ± 1.2^(c)^

Superscript letters a, b, c, and d denote a statistically significant difference at (*P* < 0.05) using one-way ANOVA with Tukey’s post hoc test

^a^Statistical significance when compared to group I

^b^Statistical significance when compared to group II

^c^Statistical significance when compared to group III

^d^Statistical significance when compared to group IV

### Effect of phoenixin treatment on mitochondrial transmembrane potential (ΔΨm), complex I of electron transport chain (ETC) and ROS among the studied groups

**Fig. 1:**
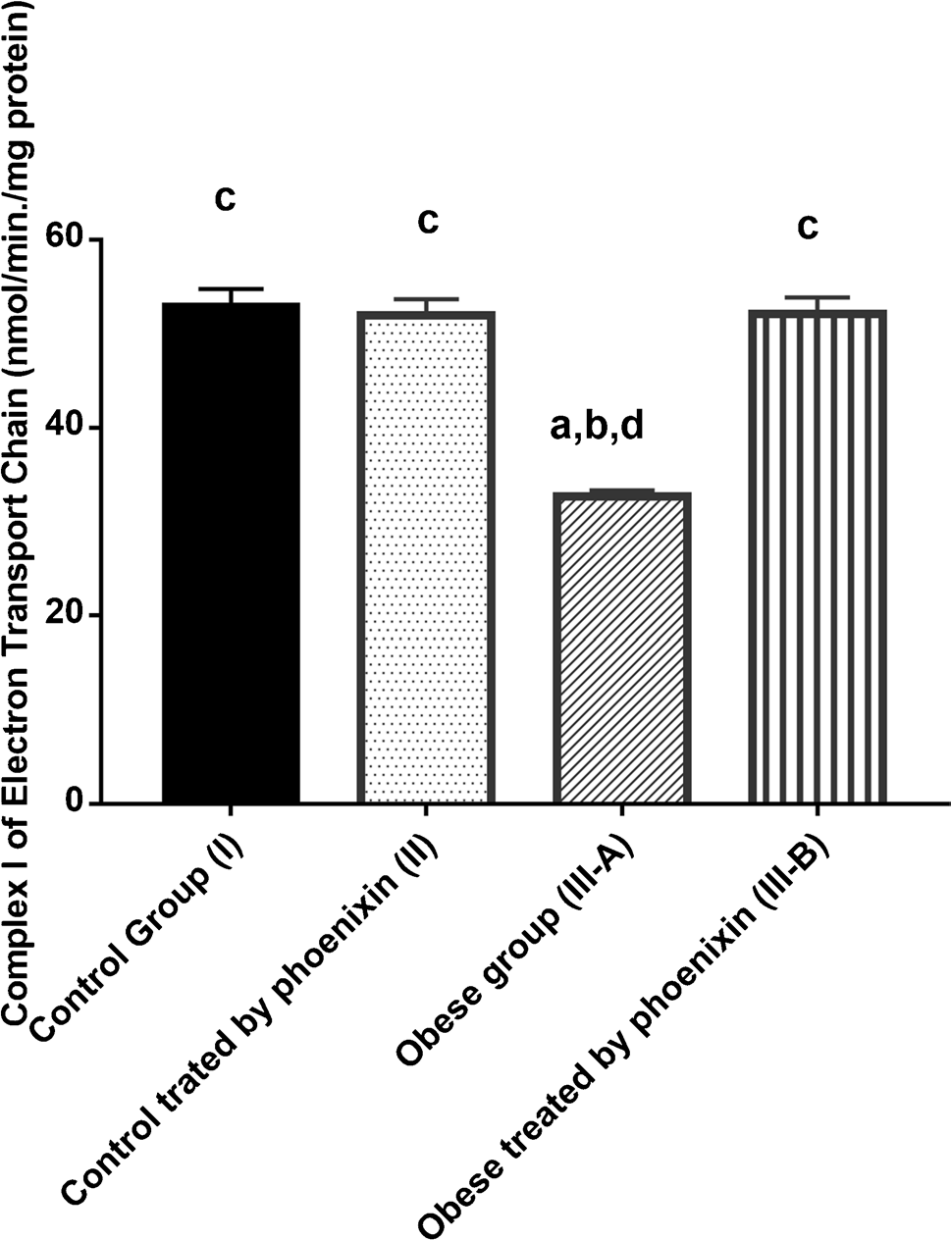
Effect of phoenixin treatment on Complex I of electron transport chain (ETC). Values are represented as mean ± SD (*n* = 20). Superscript letters a, b, c, and d denote a statistically significant difference at (*P* < 0.05). ^a^Statistical significance when compared to group I. ^b^Statistical significance when compared to group II. ^c^Statistical significance when compared to group III. ^d^Statistical significance when compared to group IV using one-way ANOVA with Tukey post hoc test

**Fig. 2:**
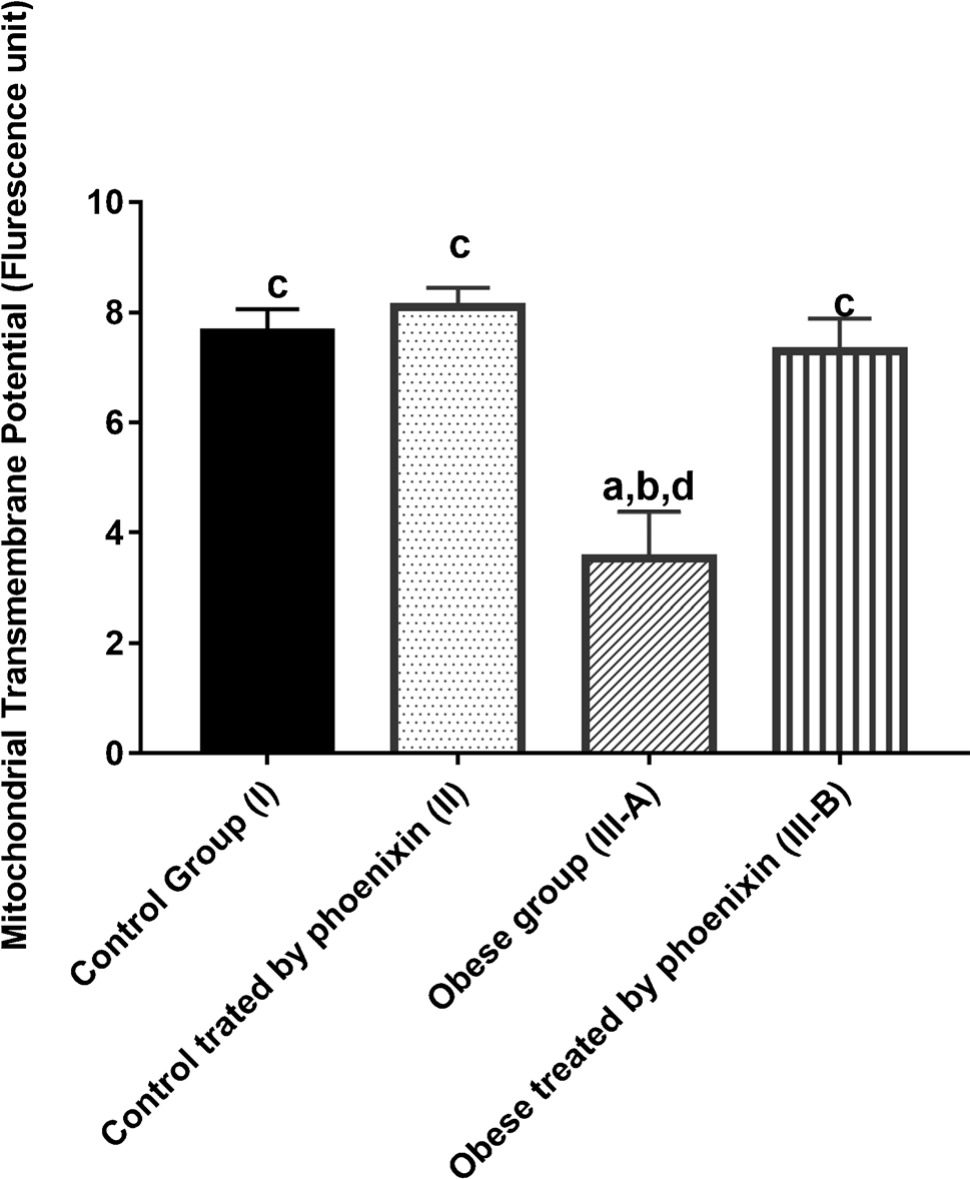
Effect of phoenixin treatment on mitochondrial transmembrane potential (ΔΨm). Values are represented as mean ± SD (*n* = 20). Superscript letters a, b, c, and d denote a statistically significant difference at (*P* < 0.05). ^a^Statistical significance when compared to group I. ^b^Statistical significance when compared to group II. ^c^Statistical significance when compared to group III. ^d^Statistical significance when compared to group IV using one-way ANOVA with Tukey post hoc test

**Fig. 3:**
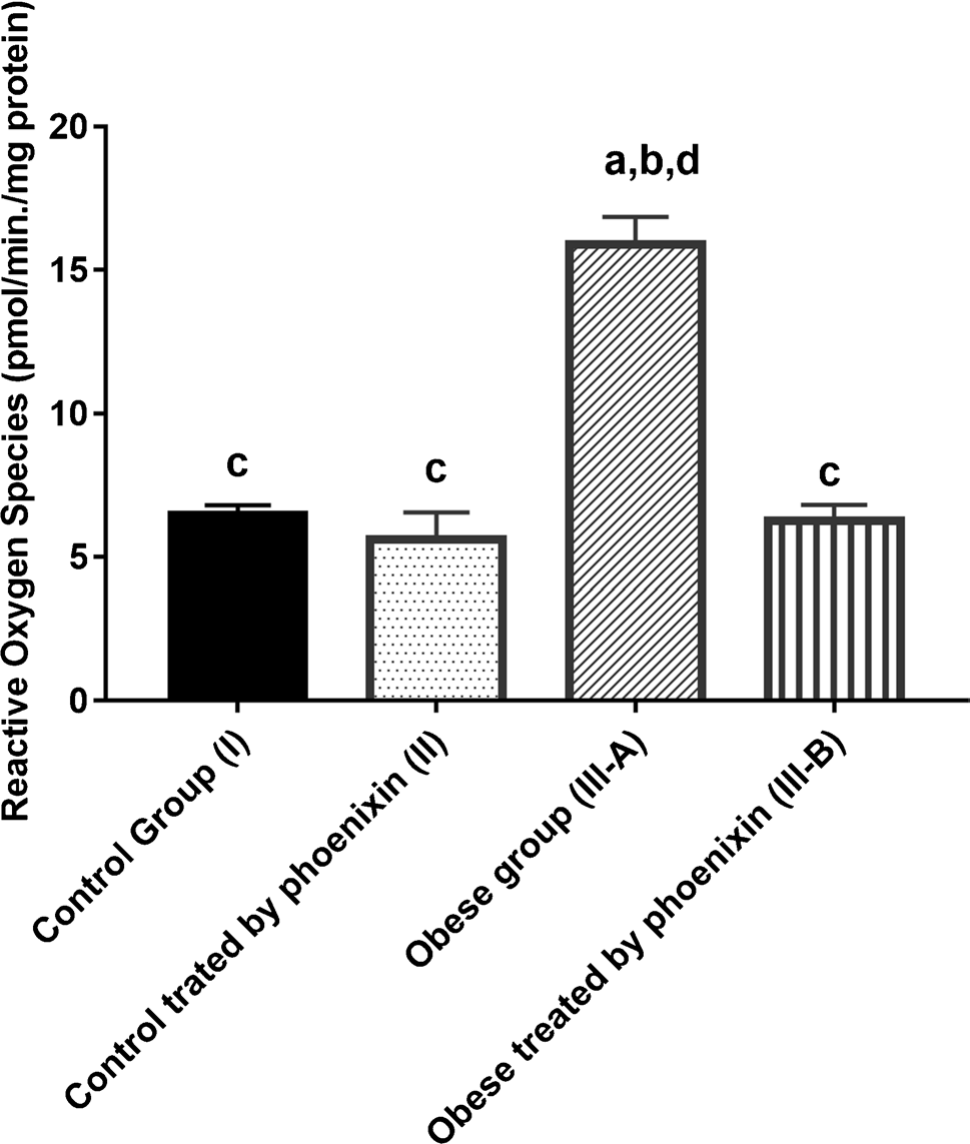
Effect of phoenixin treatment on ROS levels. Values are represented as mean ± SD (*n* = 20). Superscript letters a, b, c, and d denote a statistically significant difference at (*P* < 0.05). ^a^Statistical significance when compared to group I. ^b^Statistical significance when compared to group II. ^c^Statistical significance when compared to group III. ^d^Statistical significance when compared to group IV using one-way ANOVA with Tukey post hoc test

Table 4 shows a significant decrease in mitochondrial transmembrane potential (ΔΨm) and complex I of ETC levels in the obese group with a concomitant increase in ROS level as compared to the control groups. Treatment with PNX has corrected this significantly lowered level of (ΔΨm) and complex I and decreased the ROS level in the treated group as compared to the obese group. There is no significant difference between control groups and PNX-treated group in (ΔΨm), complex I, and ROS levels (Figs. 1, 2, 3).

**Table 4:** Effect of phoenixin treatment on mitochondrial transmembrane potential (ΔΨm), complex I of electron transport chain (ETC) and ROS

Parameter	Control group (I)^a^ *N* = 20	Control treated by phoenixin (II)^b^ *N* = 20	Obese (III)^c^ *N* = 20	Obese treated with phoenixin (IV)^d^ *N* = 20
(ΔΨm) (fluresence unit)	7.6 ± 0.42^(c)^	8.1 ± 0.34^(c)^	3.5 ± 0.85^(a, b, d)^	7.3 ± 0.59^(c)^
Complex I (nmol/min./mg protein)	52.9 ± 1.9^(c)^	51.97 ± 1.68^(c)^	32.6 ± 0.67^(a, b, d)^	52.1 ± 1.7^(c)^
ROS(pmol/min./mg protein)	6.5 ± 0.35^(c)^	5.6 ± 0.93^(c)^	15.9 ± 0.97^(a, b, d)^	6.3 ± 0.5^(c)^

Superscript letters a, b, c, and d denote a statistically significant difference at (*P* < 0.05) using one-way ANOVA with Tukey’s post hoc test

^a^Statistical significance when compared to group I

^b^Statistical significance when compared to group II

^c^Statistical significance when compared to group III

^d^Statistical significance when compared to group IV

### Effect of phoenixin on relative mRNA abundance of Drp1 and MFN2 among the studied groups

**Fig. 4:**
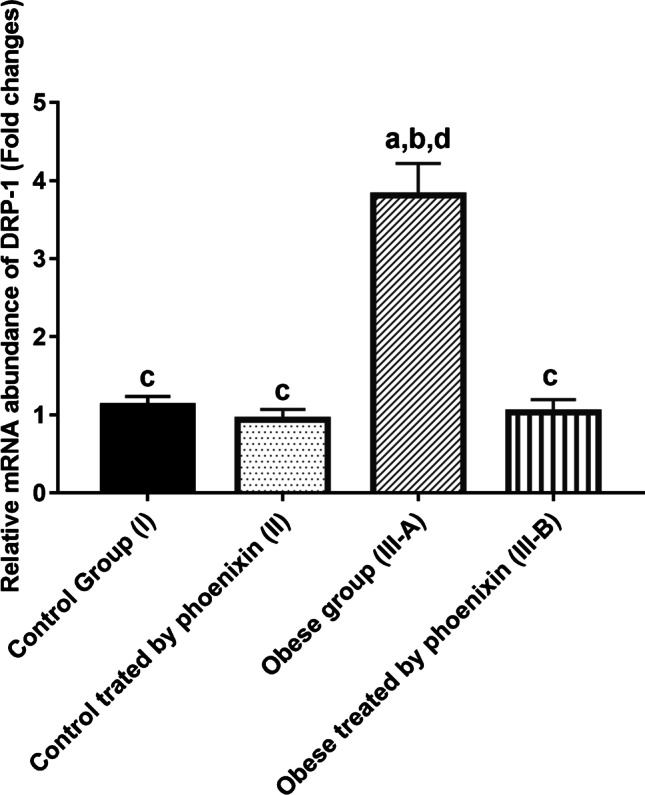
Effect of phoenixin treatment on Drp1 relative mRNA abundance. Values are represented as mean ± SD (*n* = 20). Superscript letters a, b, c, and d denote a statistically significant difference at (*P* < 0.05). ^a^Statistical significance when compared to group I. ^b^Statistical significance when compared to group II. ^c^Statistical significance when compared to group III. ^d^Statistical significance when compared to group IV using one-way ANOVA with Tukey post hoc test

**Fig. 5:**
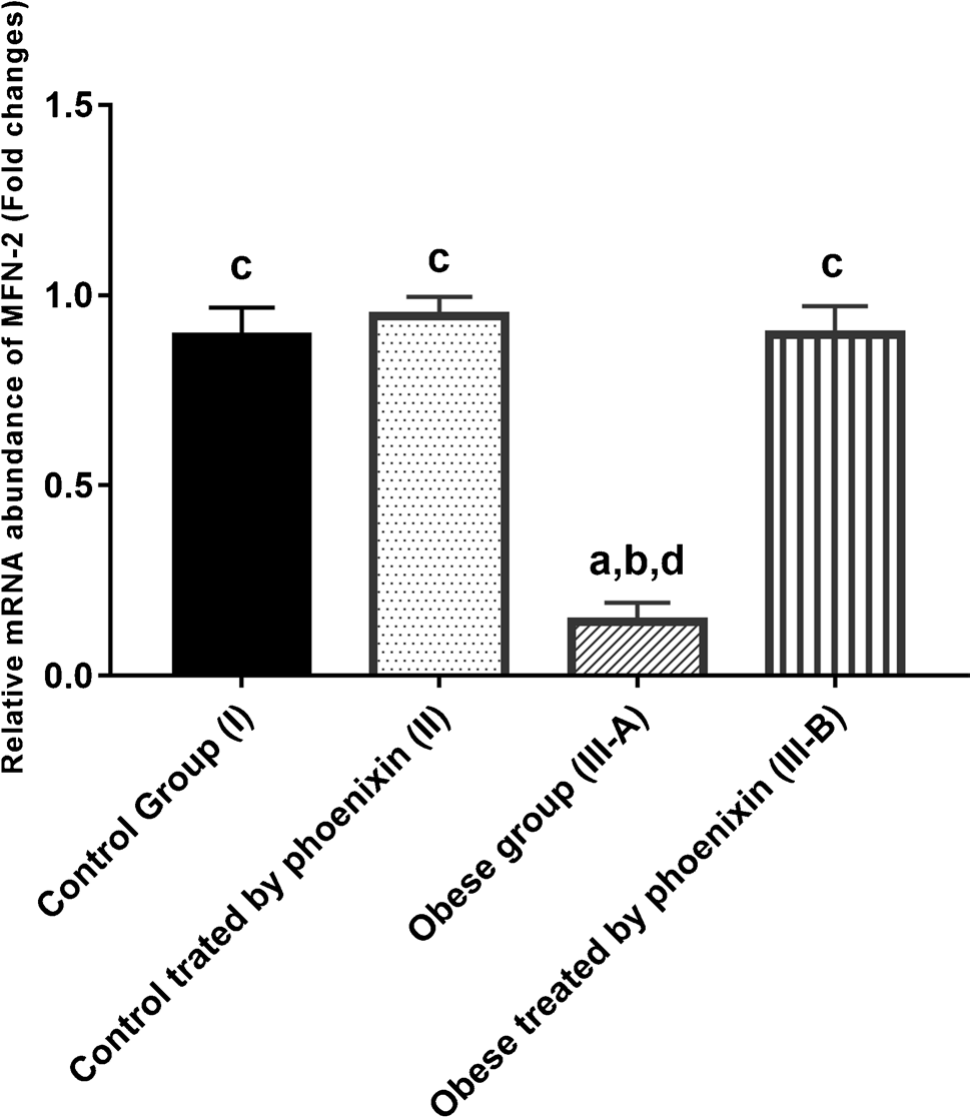
Effect of phoenixin treatment on MFN2 relative mRNA abundance. Values are represented as mean ± SD (*n* = 20). Superscript letters a, b, c, and d denote a statistically significant difference at (*P* < 0.05). ^a^Statistical significance when compared to group I. ^b^Statistical significance when compared to group II. ^c^Statistical significance when compared to group III. ^d^Statistical significance when compared to group IV using one-way ANOVA with Tukey post hoc test

The obese group showed significant upregulation of Drp1 mRNA abundance and downregulation of Mfn2 as compared to the control groups. PNX treatment has significantly corrected this imbalance, (Figs. 4, 5).

### Effect of phoenixin treatment on GnRHR relative mRNA abundance

**Fig. 6:**
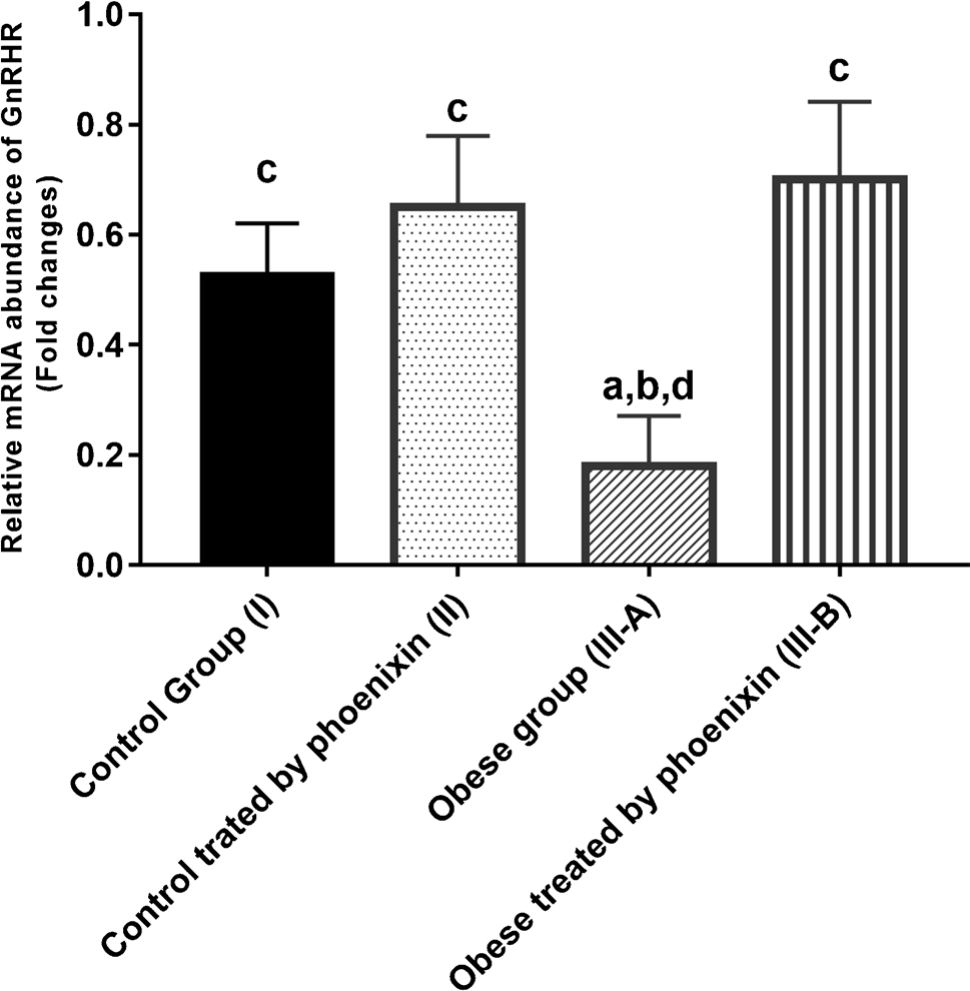
Effect of phoenixin treatment on GnRHR relative mRNA abundance. Values are represented as mean ± SD (*n* = 20). Superscript letters a, b, c, and d denote a statistically significant difference at (*P* < 0.05). ^a^Statistical significance when compared to group I. ^b^Statistical significance when compared to group II. ^c^Statistical significance when compared to group III. ^d^Statistical significance when compared to group IV using one-way ANOVA with Tukey post hoc test

The obese group showed significant downregulation of GnRHR mRNA abundance and as compared to the control groups. PNX treatment has significantly corrected this imbalance, (Fig. 6).

### Histopathology

**Fig. 7:**
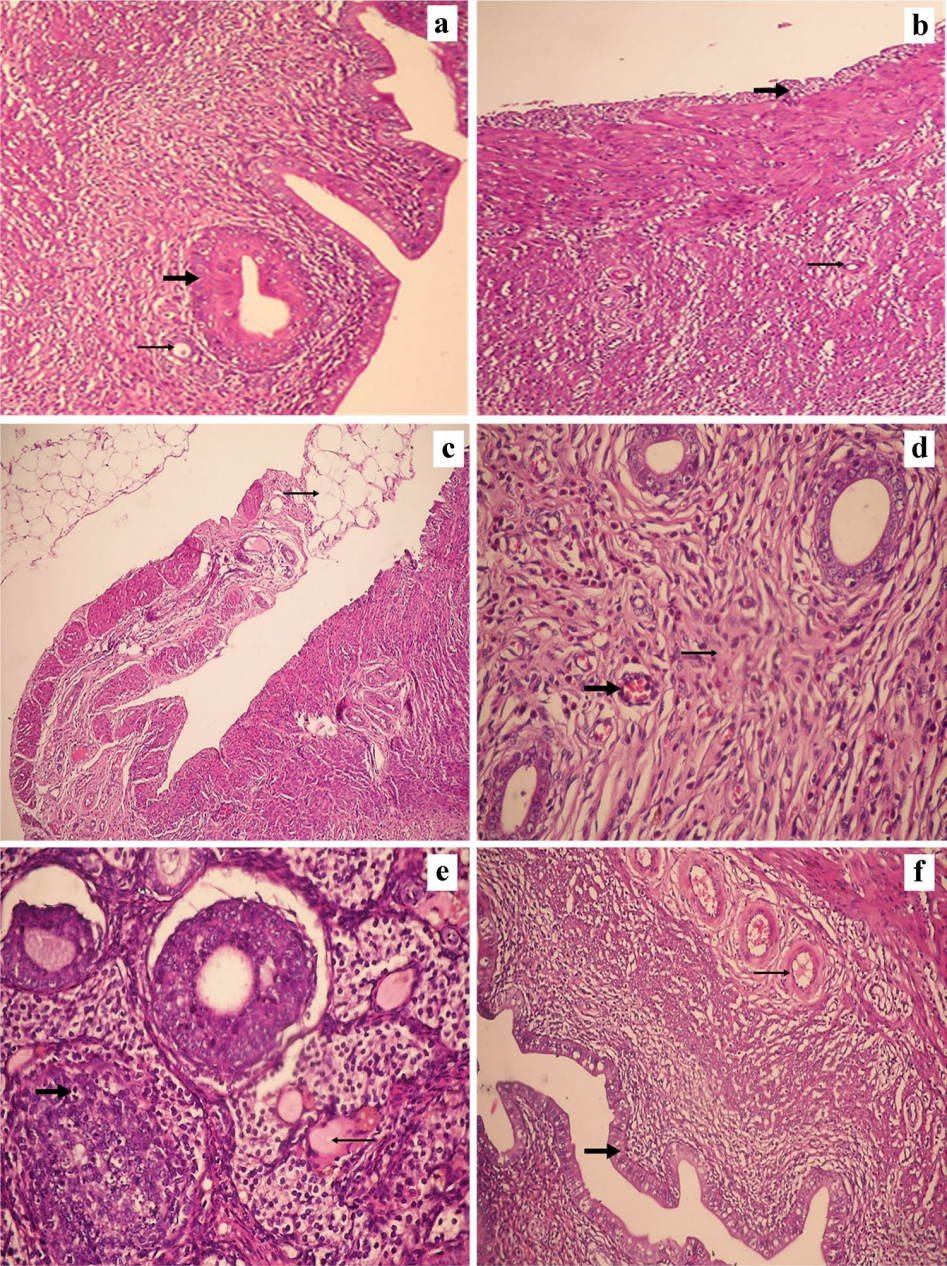
Histopathological results. **a** Section in control group (I) showing normal ovarian tissue with many primordial follicles (thin arrow) and primary follicles (thick arrow) in the ovarian cortex, covered with surface epithelium (H&E × 400), **b** section in control group (II) showing normal ovarian tissue with many primordial follicles (thin arrow), covered with surface epithelium (thick arrow), within normal ovarian stroma (H&E × 200), **c** section in obese group (III) shows increased peri-ovarian fat (thin arrow) (H&E × 100), **d** section in obese group (III) showing primordial follicles surrounded by interstitial hyalinization (thin arrow), marked interstitial inflammation, and congested blood vessels (thick arrow) (H&E × 400), **e** section in obese group (III) showing hyalinization (thin arrow) and degenerative changes of the follicular epithelium, with shrinking and pyknosis of its nuclei (thick arrow) (H&E × 400), **f** section in PNX-treated group (VI) showing normal ovarian tissue with primordial follicles (thin arrow), covered with surface epithelium (thick arrow) within loose ovarian stroma (H&E × 200)

As shown in Fig. 7, control groups (I and II) show normal ovarian tissue showing many primordial follicles (primary oocyte surrounded by a single layer of cuboidal epithelium) and primary follicles (stratified granulosa cells) in the ovarian cortex, covered with surface epithelium, within normal ovarian stroma.

Obese group (III) shows increased peri-ovarian fat and marked interstitial hyalinization. The primordial follicles are surrounded by interstitial fibrosis, marked interstitial inflammation, and congested blood vessels. The follicular epithelium shows atrophic and degenerative changes with shrinking and pyknosis of its nuclei.

PNX-treated group (VI) shows normal ovarian tissue with primordial follicles within the ovarian cortex, covered with surface epithelium within loose ovarian stroma.

## Discussion

**Fig. 8:**
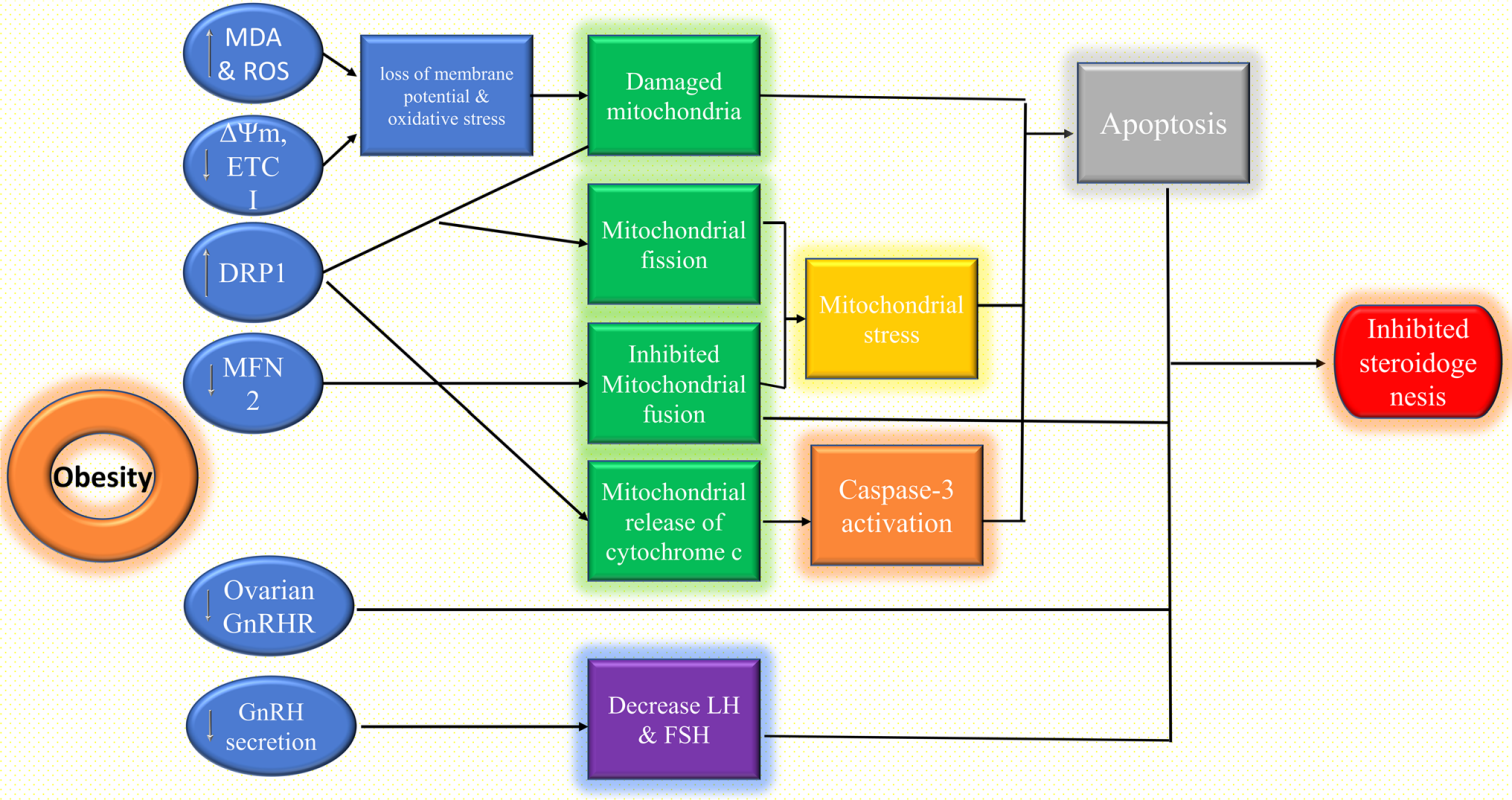
Summary scheme of the reproductive defects in obesity

The current study has revealed that PNX treatment for 10 weeks significantly decreased obesity-induced infertility in adult female rats, as confirmed by the biochemical, histopathological, and real-time gene abundance results. In addition, we investigated the effect of PNX on mitochondrial functions and its relation to ovarian cells’ survival, function, and hormonal receptor status Fig. 8.

### Effect of PNX on mitochondrial dynamics modulates obesity-induced oxidative stress

Since the cellular redox state is one of the significant controllers of cell function, gene abundance, and cell survival, we investigated the effect of PNX on oxidative stress and the potential role of mitochondrial function modulation.

Furthermore, we noted that obesity-induced alteration in mitochondrial dynamics pathways with increased mitochondrial fission as indicated by significant over-expression of the fission protein Drp1 accompanied by a decreased mitochondrial fusion as indicated by the substantial reduction in expression of fusion protein Mfn2 with the subsequent imbalance between mitochondrial fusion and fission. This finding can be attributed to obesity generating the fission process utilizing Drp1 recruitment from the cytosol to the dysfunctional site to cleave the damaged mitochondrial site. When mitochondrial damage is induced by the loss of membrane potential or oxidative stress [28], which developed in the current study, as evidenced by the significantly decreased ΔΨm, ETC complex I, and increased MDA and ROS in obese rats.

Broughton and Jungheim [3] reported that the increased circulating levels of FFA in obese women induce increased ROS with subsequent mitochondrial stress culminating in apoptosis in variable tissue types, including ovarian tissue. These results are consistent with the report by Sebastián et al. [34] that obese subjects demonstrate inhibited Mfn2 associated with decreased substrate oxidation, cellular metabolism, and reduced membrane potential in ETC complexes. Furthermore, obesity caused a reduction in mitochondrial O2 respiration and ATP production. Lipid accumulation stimulated mitochondrial ROS emission as H2O2 emission [11]. It was observed that PNX treatment significantly improved the mitochondrial dysfunction induced by obesity. Mitochondria, particularly the ETC, is a vital controller of the cell’s ROS production. PNX treatment restored the cell’s redox state to control levels, as evident by the PNX-induced significant increase of complex I ETC and Δψm levels with a subsequent decrease in ROS levels. Consistent with our results, Ma et al. [19] have demonstrated the PNX-14’s role in relieving oxidative stress and increasing glutathione levels in ischemia.

Interestingly, PNX treatment also restored balanced mitophagy to almost a control level. This finding was achieved by decreasing mitochondrial fission, as evidenced by the significantly reduced abundance of the Drp1 gene, and increased mitochondrial fusion, as evidenced by the elevated abundance of the Mfn2 gene.

### The role of PNX-induced modulation of mitochondrial functions and upregulated expression of GnRHR in promoting ovarian cell survival and decreased apoptosis

Obesity-induced infertility is marked by ovarian cell apoptosis, as evidenced by our histopathological results and the significant increase in caspase-3 activity. Obesity increases the mitochondrial release of cytochrome c to the cytoplasm due to impaired mitochondrial balance between pro-apoptotic proteins (Bax) and anti-apoptotic proteins (Bcl-2, Bcl-XL). The released cytochrome c binds to apoptotic protease-activating factor-1 (Apaf1), dATP, and pro-caspase-9, activating caspase-3 resulting in DNA fragmentation, which is a hallmark of apoptosis [11].

Bcl-2 family are also reported as regulators of mitochondrial fusion and fission through interacting with Mfn2 and Drp-1 [33]. DRP1 accumulation was reported to mediate and precede cytochrome c release with subsequent caspase activation and apoptosis. Consequently, increased mitochondrial fission usually occurs before apoptosis develops. In contrast, overexpression of Mfn2 delays Bax activation, cytochrome c release, and apoptosis [38]. This suggested a strong link between PNX-induced regulation of mitochondrial dynamics and enhanced the ovarian cell survival observed in our histopathological results, and decreased caspase-3.

Our histopathological results demonstrated an antiapoptotic effect of PNX treatment on ovarian tissue, which is consistent with the results of Nguyen et al. [25] who reported that PNX and its receptor successfully promoted the growth of ovarian follicles in vitro, which has proven to have a dose-dependent pattern by Komatsu et al. [16] and Murase et al. [24].

Consistent with our results, PNX was reported to decrease myocardial apoptosis by blocking the upregulation of pro-apoptotic genes, such as Bax and caspase 3, and increasing the abundance of the anti-apoptotic gene, Bcl-2, possibly through the reperfusion injury salvage kinase (RISK) and survival activating factor enhancement (SAFE) pathways. RISK activates pro-survival kinases that protect the heart by inhibiting apoptosis, while SAFE acts through STAT3 [21].

Moreover, the respiratory chain affects apoptosis. Respiratory chain dysfunction enhanced ROS production and changed the complement of anti-apoptotic proteins in the mitochondria [17]. Remarkably, Δψm is also crucial for cell maintenance, which was also reported by Wu et al. [43] who illustrated that reduced cell viability and Δψm caused by obesity were shown to be almost completely ameliorated by PNX-14 treatment, which is also evident in the current study. This furtherly emphasizes that PNX-induced modulation of mitochondrial functions is one of the proposed mechanisms for enhancing ovarian cell survival.

On the contrary, the current study also showed upregulated expression of GnRHR. GnRH was reported to protect ovarian tissue and promote ovarian cell survival, especially with cytotoxic drugs. Although not yet established, upregulated ovarian GnRHR was suggested to enhance ovarian follicle survival probably through the sphingosine-1 phosphate-dependant mechanism [36]. This finding indicates that PNX-induced upregulation of GnRHR also promoted ovarian cell survival.

### Role of PNX in hormonal profile modulation

The current study results demonstrated that the obese group rats showed significantly increased insulin levels along with obesity, as indicated by the significant increase in cholesterol and triglycerides levels. They also developed hormonal impairment as indicated by the significant decrease in estrogen, progesterone, LH, and FSH.

The developed hyperinsulinemia and insulin resistance is a condition accompanying obesity that was attributed to various factors, including free fatty acids, leptin, cytokines, and androgens [29]. Hyperinsulinemia induced an increase of local androgen production by theca cells either directly or by increasing theca cells’ sensitivity to LH along with the action of ovarian insulin-like growth factor-I (IGF-I) on its ovarian receptors [15, 35]. Increasing intra-ovarian androgen consequently induced premature follicular atresia, which is evident in our histopathological results, with subsequent anovulation, which explains hyperinsulinemia-induced infertility.

Obesity, in addition to intra-ovarian hyperandrogenism, caused increased tissue targeting by free blood androgen secondary due to the combined effect of the obtained regarding increased adipose tissue synthesis of androgens and decreased sex hormone-binding globulin (SHBG) circulating levels described by [8].Obese women’s ovaries also show apoptosis in granulosa cells, which could be attributed to increased androgen [4].

Furthermore, increased androgen peripheral conversion to estrogens in adipose tissue deteriorates the function of the hypothalamic-pituitary–gonadal (HPG) axis with subsequent inhibition of gonadotropin secretion which furtherly contributes to hormonal imbalance, anovulation, and fertility impairment.

In contrast, PNX treatment demonstrated a corrected hormonal profile to almost a control level, which could be due to the fact that PNX acts on both central and local intracellular levels to enhance ovarian steroidogenesis eventually.

### The role of PNX-induced mitochondrial dynamics modulation and upregulation of GnRH receptor in local improvement of ovarian steroidogenesis

In the ovary, reorganization of cellular organelles and contact of membranes is necessary for hormone synthesis and secretion since many enzymes are localized between mitochondria and the endoplasmic reticulum, highlighting the necessity of mitochondrial fusion for normal hormonal levels to be obtained [7]. This finding explains that the PNX-induced Mfn2 upregulation, as demonstrated in the current study, is a proposed mechanism for restoring ovarian hormone levels.

Also, Park et al. [14] reported that Drp-1 phosphorylation determines mitochondrial shaping inducing mitochondrial elongation through inhibiting mitochondrial fission, which offers an optimal environment for steroidogenesis. Interestingly, this phosphorylation process is mediated by a cAMP/PKA pathway [25] similar to the pathway activated by ovarian GPR173 stimulation, suggesting that GPR173 activation may induce Drp1 phosphorylation and decrease the activity of cytoplasmic Drp1 alongside its effect inhibiting its protein synthesis.

Furthermore, mitochondria are dynamically distributed using cytoskeletal tracks and motor protein for their intracellular relocation. It is reported that mitochondria are arrested at the sites of Ca^+2^ elevation, providing the sufficient energy supply for the processes where Ca + 2 is used to signal for [10]. As a G-protein-coupled receptor, GnRHR increases intracellular Ca + 2 by activating phospholipase C, we suggest retaining the mitochondria at the upregulated GnRHR signaling sites, which might be related to steroid biosynthesis, as another proposed mechanism for increasing ovarian steroidogenesis back to the normal levels after PNX treatment.

Also, Duarte et al. [7] illustrated that hormonal stimulation by hCG triggers mitochondrial fusion into tubular-shaped structures to meet the increasing demand for energy during steroidogenesis. Given the previously described cytoprotective role of GnRH in the ovary, combined with the PNX-induced overexpression of GnRHR, described in the current work, we proposed that GnRH can have a role in regulating mitochondrial fusion to relocate the energy production sites within the course of its cytoprotective effect in the ovary. In contradiction with our explanation, Hom et al. [12] described the role of intracellular Ca^+2^, which can be induced by GnRHR stimulation, in increasing Drp-1 with more mitochondrial fission in cardiomyocytes. Also, Metallinou et al. [23] described GnRH-induced increase in Drp-1 in gonadal cells inducing apoptosis. These discrepancies can be justified by the different targeted cells or the different duration and doses of exposure to exogenous GnRH or its analogues, or the different mechanisms of action of GnRHR in these cases which necessitates further investigations of this point.

Also, in support of our results, PNX-20 was found to upregulate critical gene expression in gonadal sex steroidogenesis peripherally in both male and female zebrafish (Rajeswari & Unniappan, 2020).

Therefore, apart from its previously reported role in increasing GnRH secretion and sensitization of pituitary to GnRH, also denoted in our study by the PNX-induced increase in LH and FSH, we report that PNX increased ovarian sensitivity to circulating GnRH by inducing upregulated expression of GnRHR. Together with the modulation of mitochondrial functions, these are proposed mechanisms for the local effect of PNX on ovarian steroidogenesis.

### Central role of PNX in improving the hormonal profile

The results of Wang et al. possibly rationalize the improvement of the hormonal profile by PNX [41] who reported that PNX treatment directly stimulates both the HPG-axis and the pituitary gland. PNX-20 stimulates the release of GnRH-induced LH, which is probably mediated by PNX-stimulated GnRH receptor upregulation [37]. This can be induced either by central or peripheral administration of PNX since it can cross the blood–brain barrier.

Furthermore, kisspeptin system overexpression was reportedly induced by PNX-20 treatment, which denotes PNX-20’s evident influence on the hypothalamic regulation of reproduction [26]. It was also reported that in the rat hypothalamus, PNX stimulates kisspeptin-1 transcription in both arcuate and anteroventral periventricular (AVPV) nucleus 3 [39].

Regarding the anti-inflammatory effect of PNX shown in our results, Yao et al. [46] reported the role of PNX-14 in the downregulation of the expression of obesity-induced cytokines. Furthermore, PNX-14 may significantly inhibit the HMGB1/TLR4/MyD88/NF-κB inflammatory cascade by downregulating the expression and activation of each of these factors. Also, the anti-inflammatory functions of GnRH may involve PNX as an anti-inflammatory compound, suggesting a therapeutic role for PNX in inflammatory diseases [18].

## Conclusion

We conclude that phoenixin possesses a significant role in the positive modulation of obesity-induced fertility impairment by acting locally on mitochondrial machinery and GnRHR gene upregulation together with its well-known central effect. The peripheral role of phoenixin in such conditions comprises not only the systemic effect on ameliorating obesity, but also its highlighted local gonadal improvement of mitochondrial dynamics and upregulation of GnRHR mRNA abundanc which are suggested to improve ovarian cellular resistance to obesity-induced inflammatory, oxidative, and apoptotic stress leading to better cell survival and steroidogenesis.

## Recommendations

Our data suggest that phoenixin can be used as a promising adjuvant therapy in the treatment of obesity-induced fertility impairment. However, further research is required to fully understand its safety limits and toxicity, verify its effect on the ovarian protein content of DRP-1, MFN-2, and GnRHR rather than their mRNA abundance, and study its effect on ovarian gene expression of gonadotropin receptors in obese females and its effect in other cases of fertility impairment such as polycystic ovary syndrome. Also, the expression of phoenixin and its receptor in obese infertile subjects both centrally and in the gonads need further research to obtain a better understanding of the role of phoenixin in such conditions. More work is also needed to investigate the possible link between the cytoprotective role of PNX-induced GnRH sensitization and mitochondrial dynamics.

## Data Availability

All data that support the finding of the current study are available from R.A.E.G., upon reasonable request.
